# Monitoring Sleep Quality Through Low α-Band Activity in the Prefrontal Cortex Using a Portable Electroencephalogram Device: Longitudinal Study

**DOI:** 10.2196/67188

**Published:** 2025-03-10

**Authors:** Chuanliang Han, Zhizhen Zhang, Yuchen Lin, Shaojia Huang, Jidong Mao, Weiwen Xiang, Fang Wang, Yuping Liang, Wufang Chen, Xixi Zhao

**Affiliations:** 1 School of Biomedical Sciences and Gerald Choa Neuroscience Institute The Chinese University of Hong Kong Hong Kong China (Hong Kong); 2 Department of Mathematics and Statistics University of Massachusetts at Amherst Amherst, MA United States; 3 Shenzhen Institute of Advanced Technology Chinese Academy of Sciences Shenzhen China; 4 Shenzhen Shuimu AI Technology Co., Ltd Shenzhen China; 5 National Clinical Research Center for Mental Disorders & National Center for Mental Disorders, Beijing Key Laboratory of Mental Disorders Beijing Anding Hospital Capital Medical University Beijing China; 6 Advanced Innovation Center for Human Brain Protection and Laboratory for Clinical Medicine, Capital Medical University Beijing China

**Keywords:** EEG, electroencephalogram, alpha oscillation, prefrontal cortex, sleep, portable device

## Abstract

**Background:**

The pursuit of sleep quality has become an important aspect of people’s global quest for overall health. However, the objective neurobiological features corresponding to subjective perceptions of sleep quality remain poorly understood. Although previous studies have investigated the relationship between electroencephalogram (EEG) and sleep, the lack of longitudinal follow-up studies raises doubts about the reproducibility of their findings.

**Objective:**

Currently, there is a gap in research regarding the stable associations between EEG data and sleep quality assessed through multiple data collection sessions, which could help identify potential neurobiological targets related to sleep quality.

**Methods:**

In this study, we used a portable EEG device to collect resting-state prefrontal cortex EEG data over a 3-month follow-up period from 42 participants (27 in the first month, 25 in the second month, and 40 in the third month). Each month, participants’ sleep quality was assessed using the Pittsburgh Sleep Quality Index (PSQI) to estimate their recent sleep quality.

**Results:**

We found that there is a significant and consistent positive correlation between low α band activity in the prefrontal cortex and PSQI scores (*r*=0.45, *P*<.001). More importantly, this correlation remained consistent across all 3-month follow-up recordings (*P*<.05), regardless of whether we considered the same cohort or expanded the sample size. Furthermore, we discovered that the periodic component of the low α band primarily contributed to this significant association with PSQI.

**Conclusions:**

These findings represent the first identification of a stable and reliable neurobiological target related to sleep quality through multiple follow-up sessions. Our results provide a solid foundation for future applications of portable EEG devices in monitoring sleep quality and screening for sleep disorders in a broad population.

## Introduction

Sleep is a fundamental behavior closely related to daily life, and sleep quality plays a crucial role in human health [1-4]. Poor sleep quality often leads to various types of physical ailments [5-7]. Previous studies have conducted systematic research across different species (flies [8], rodents [9-14], cats [15,16], nonhuman primates [17], and humans [18-21]) to characterize the neural mechanisms across multiple stages of sleep. However, there are still many challenges in translating findings from animal experiments to humans, and sleep remains a complex concept. Our understanding of the relationship between sleep quality and the brain is still incomplete.

Electroencephalogram (EEG) is widely used in human studies to investigate sleep-related issues [22-24]. Different types of neural oscillations typically represent different stages of sleep [10] and are applied in various research areas. However, sleep quality is a subjective experience, and we hypothesize that this subjective perception stems from the brain’s (eg, prefrontal cortex) daily neural responses. If this hypothesis holds, then under resting-state conditions, we should be able to identify certain neurobiological characteristics in the brain that are significantly associated with subjective sleep quality. Neural oscillations [25,26] are prominent biological features in EEG [27-31], such as α oscillations (8-13 Hz). Previous studies have explored the association between various types of neural oscillations and sleep quality [32-37]. However, much of the current research progress relies on single-session data collection, and there is a lack of follow-up studies. Hence, the stability of these associations remains unclear.

Therefore, in this study, to test the above hypothesis, we used a portable EEG device to collect resting-state EEG data (both open-eye and closed-eye conditions) from participants while also assessing their sleep quality (Pittsburgh Sleep Quality Index [PSQI]). The study involved a 3-month follow-up, with a total of 42 participants. In the first month, 27 people participated, followed by 25 people in the second month, and 40 people in the third month. A total of 22 participants completed all 3 sessions. We performed spectral analysis and correlation analysis on the data and further explored the periodic and aperiodic components of the spectra.

## Methods

### Participants

A total of 42 right-handed healthy individuals (19 females and 23 males; age: 34.9, SD 8.0 years) were recruited for this study, who were mainly from nearby communities through posters posted on the publicity walls. The sample size was calculated by G*Power software (Heinrich-Heine-Universität Düsseldorf), the sample size is calculated with 28 (given effect size of 0.65, α of 0.05, and β of 0.05 in the difference between means [matched pairs in eyes-open and eyes-closed state]). Hence, on average, we recruited around 30 participants each month. In the first month, 27 people participated, followed by 25 people in the second month, and 40 people in the third month. A total of 22 participants participated in all 3 months, 24 people participated in months 1 and 2, 25 people participated in months 1 and 3, and 23 people participated in months 2 and 3.

### Ethical Considerations

Informed consent was obtained from all participants. All procedures were performed according to the National Institutes of Health Guidelines, and the research protocol was approved by the Shenzhen Institute of Advanced Science and Technology, Chinese Academy of Science (SIAT-IRB-240915-H0913). The study data are anonymous. The participant compensation is calculated based on the duration of the experiment (US $14.1 per hour) each month. The timer starts when the participant begins preparation for the experiment and stops when the experiment ends (after EEG data collection and completion of the PSQI questionnaire). The total duration of each experiment is typically 15-20 minutes.

### Neurophysiological Recording

Each month, resting-state EEG in both open-eye and closed-eye states (counterbalanced order) was recorded (3 minutes each) using a 2-electrode (FP1 and FP2) setup (Brain Pro, Shenzhen Shuimu AI Technology Co Ltd, China). During the recording, the reference electrode was set at the earlobe, and real-time filtering was implemented with a bandpass filter with cutoff frequencies of 0.5 and 30 Hz. EEG data are wirelessly transmitted to a computer via Bluetooth. Each participant collected data in the morning, usually 2-3 hours after waking up. The sampling rate was 512 Hz and the scalp impedance was maintained below 10 kΩ for the electrode. Eye-blink artifacts were removed with unsupervised machine-learning algorithms [38].

### Estimation of Sleep Quality

The PSQI [39] was used to evaluate the participants’ sleep quality after monthly EEG recordings. The evaluation included several indicators: sleep quality, time to fall asleep, sleep duration, sleep disorders, daytime dysfunction, and total sleep score. Each factor is scored in 4 levels based on a score of 0-3. The cumulative score for each factor was summed to yield the PSQI total score (0-21 points), with a higher total score indicating poorer sleep quality.

### Power Spectrum Analysis

**Figure 1:**
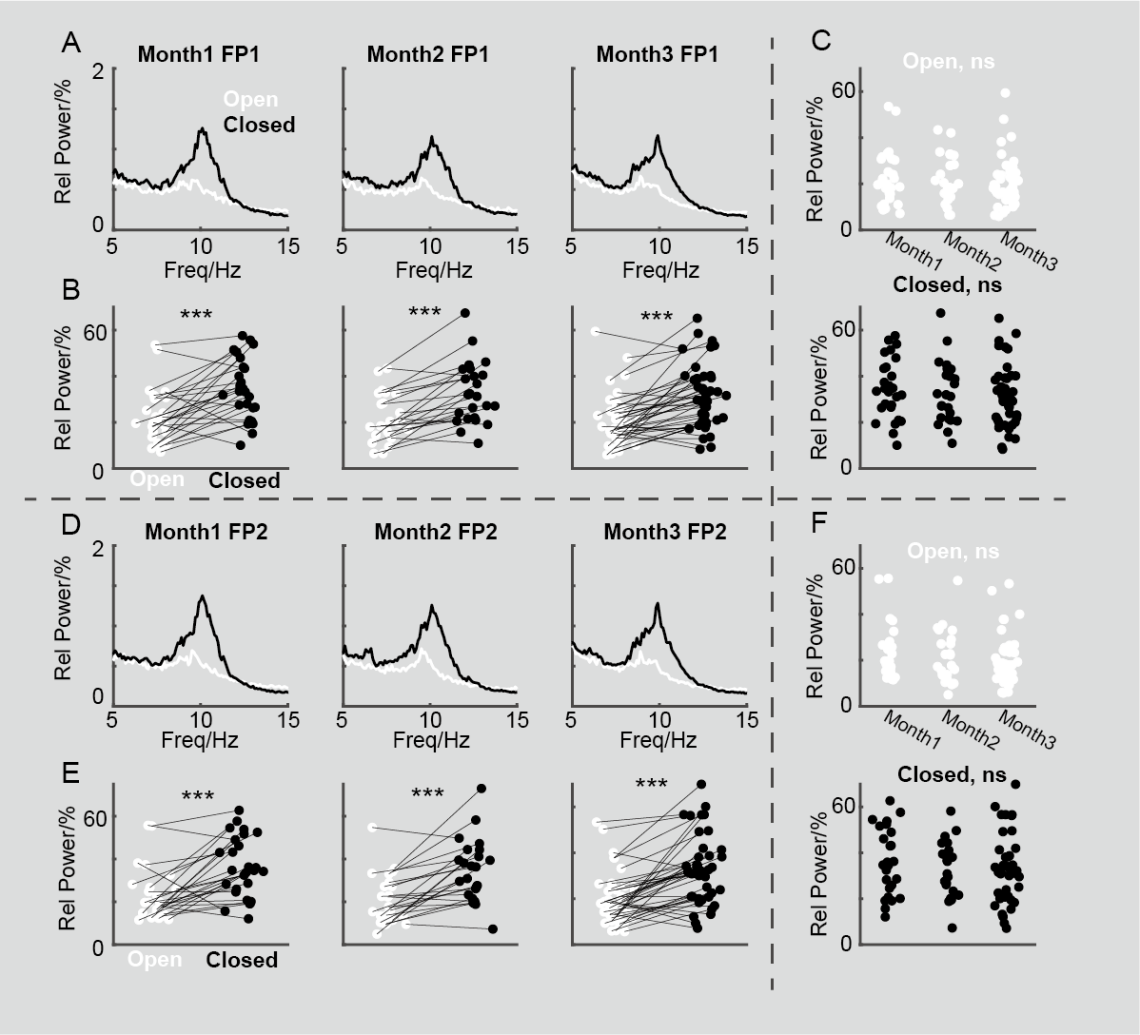
Three-month follow-up electroencephalogram recording using portable device. (A) Averaged power spectrum in FP1 during months 1, 2, and 3 under both open-eye and closed-eye states. (B) Pairwise comparison of relative α power in FP1 between open-eye and closed-eye states in months 1, 2, and 3, with *** indicating *P*<.001. (C) Comparison of relative α power in FP1 between months 1, 2, and 3 in open-eye and closed-eye states, respectively. (D) Averaged power spectrum in FP2 during months 1, 2, and 3 under both open-eye and closed-eye states. (E) Pairwise comparison of relative α power in FP2 between open-eye and closed-eye states in months 1, 2, and 3, with *** indicating *P*<.001. (F) Comparison of relative α power in FP2 between months 1, 2, and 3 in open-eye and closed-eye states, respectively.


The power spectrum of the EEG response was estimated using the multitaper method [40,41] (time-bandwidth product, 3; tapers, 5; Chronux toolbox, which was implemented using custom software written in MATLAB. Similar methods have been used in several studies in the field of biomedical science (eg, biological science [42-45], medical science [28,30], and public health [46-50]). Relative power can be defined as Equation 1 (Figure 1C).

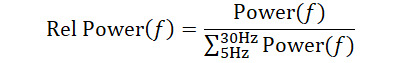
(1)


### Descriptive Model for Dissecting Aperiodic and Periodic Activity

The power spectrum was considered as the sum of 2 components: aperiodic and periodic components. Aperiodic activity in the power spectrum refers to the frequency components of brain activity that do not follow a regular periodic or oscillatory pattern. It does not have a clear, repeating waveform or regular frequency. This activity is typically more broadband and can be described by a continuous distribution of power across a range of frequencies. Periodic activity in the power spectrum refers to frequency components of brain signals that exhibit a regular, repetitive oscillatory pattern over time. These oscillations have specific, identifiable peaks in the power spectrum at distinct frequencies. The aperiodic component of the power spectrum was extracted using a 1/f-like function (Equation 2), where a, b, c, and d represent the model parameters to be estimated. The periodic component was defined as the residual obtained by subtracting the aperiodic component from the raw power spectrum. This method has been used in the characterization of γ-band and α-band activities [51-53].



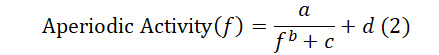



### Statistical Analysis

**Figure 2:**
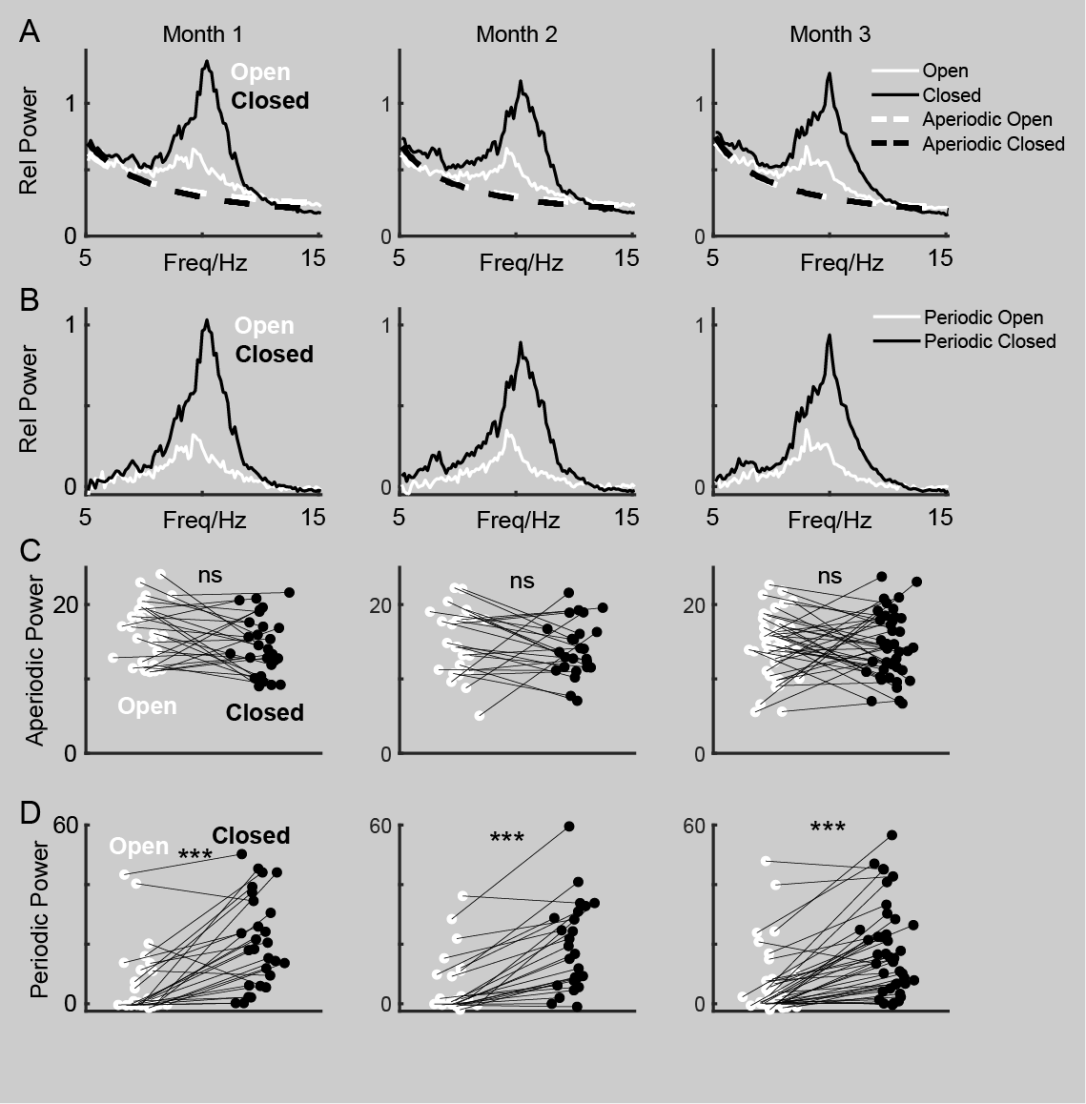
Dissection of aperiodic and periodic activity. (A) Averaged raw power spectrum showing aperiodic activities in both open-eye and closed-eye states over a 3-month period. (B) Averaged periodic activities in both open-eye and closed-eye states over a 3-month period. (C) Pairwise comparisons of relative α power in aperiodic activity between open-eye and closed-eye states in months 1, 2, and 3. (D) Pairwise comparisons of relative α power in periodic activity between open-eye and closed-eye states in months 1, 2, and 3, with *** indicating *P*<.001.

**Figure 3:**
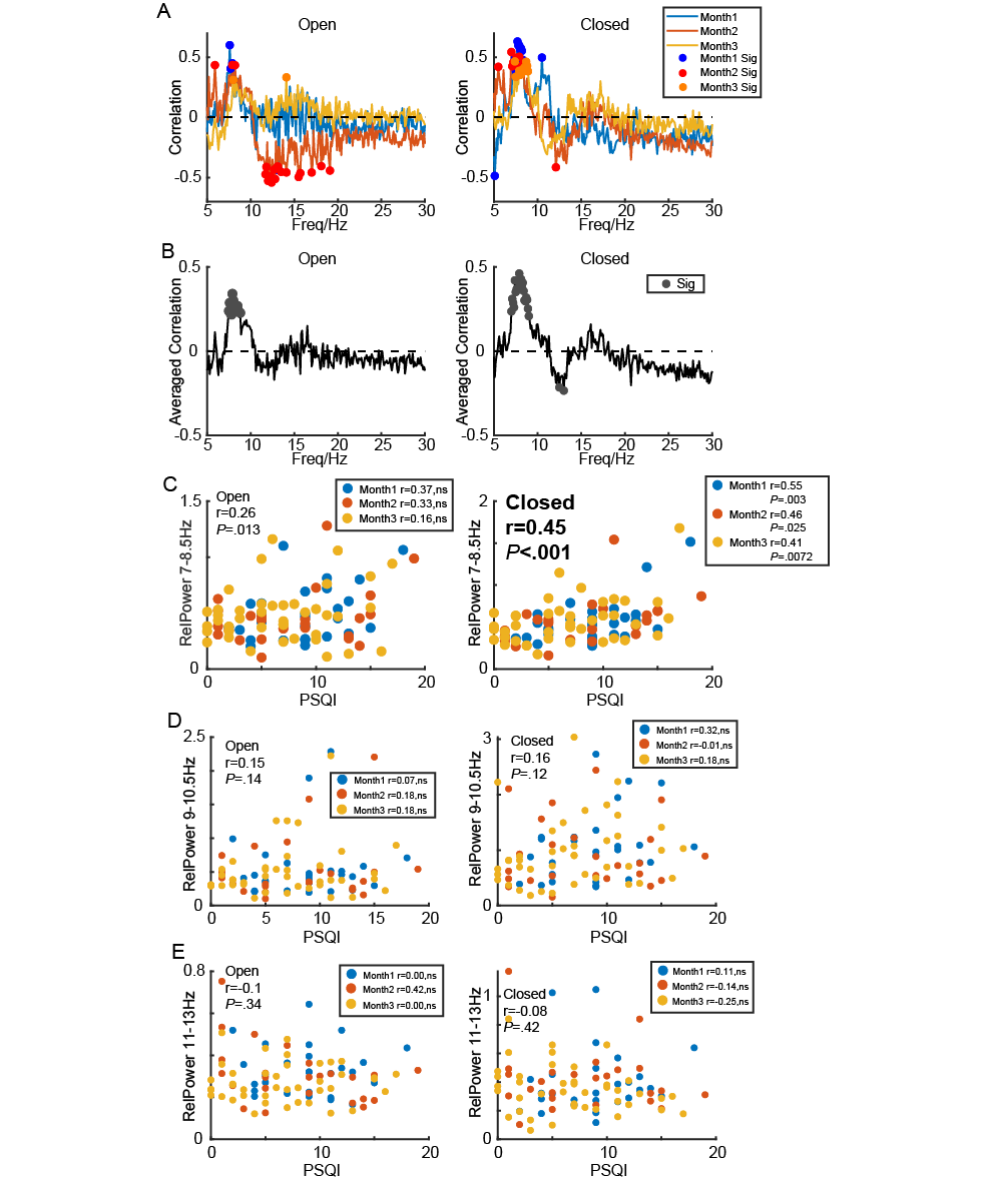
Relationship between PSQI and strength of sub-α oscillations for all participants. (A) Correlation between PSQI and relative power in difference frequencies under open-eye and closed-eye states over 3 months for all participants. The dot indicates significance at *P*<.05. (B) Averaged 3-month correlation between PSQI and relative power in difference frequencies under open-eye and closed-eye states. The dot indicates significance at *P*<.05. (C) Scatter plot showing the relationship between low α power in both open-eye and closed-eye states and PSQI for all participants in months 1, 2, and 3. (D) Scatter plot showing the relationship between medium α power in both open-eye and closed-eye states and PSQI for all participants in months 1, 2, and 3. (E) Scatter plot showing the relationship between high α power in both open-eye and closed-eye states and PSQI for all participants in months 1, 2, and 3. PSQI: Pittsburgh Sleep Quality Index.

**Figure 4:**
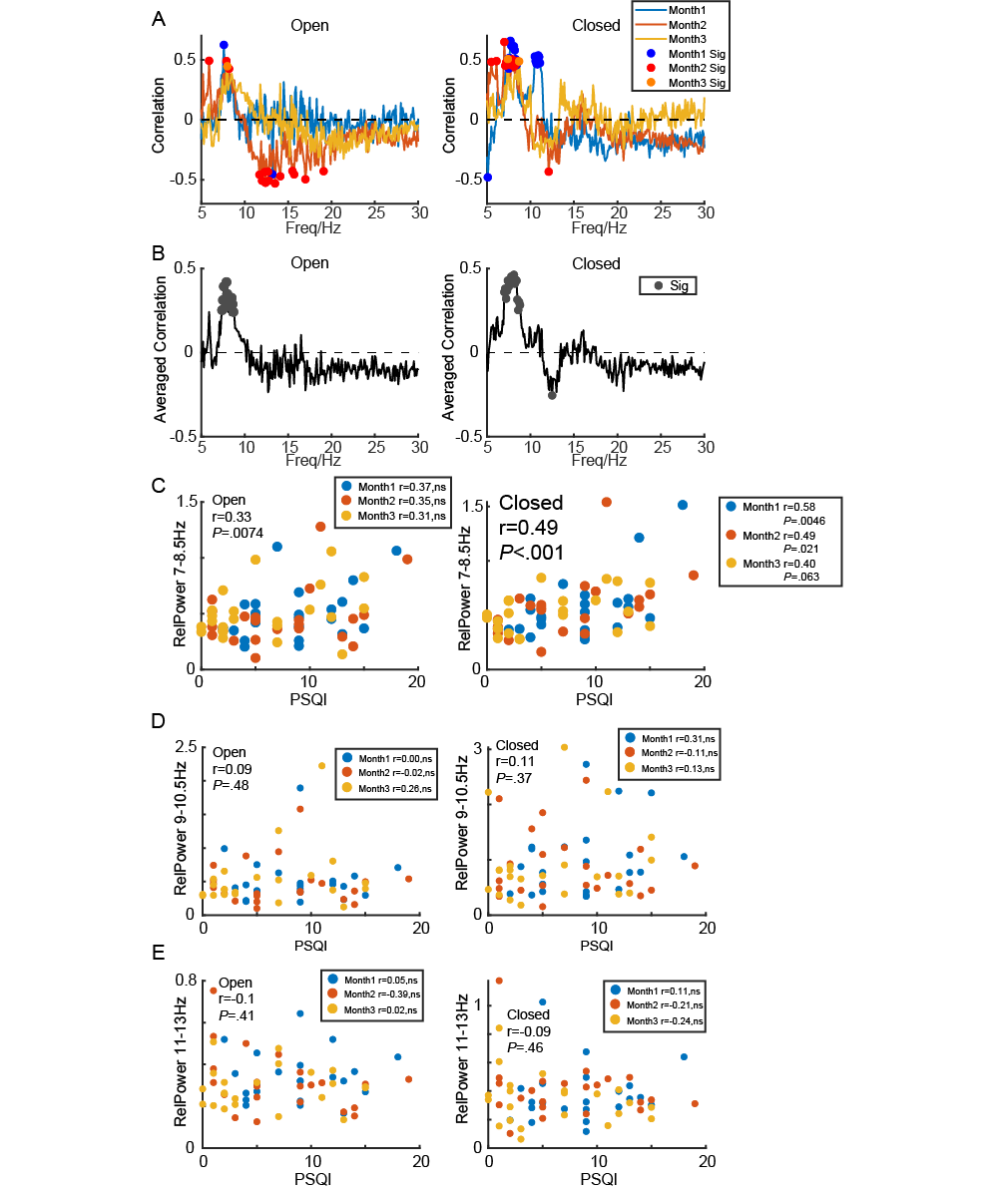
Relationship between PSQI and strength of sub-α oscillations for participants who participated in the all 3-time experiment. (A) Correlation between PSQI and relative power in difference frequencies in open-eye and closed-eye states in 3 months for participants who participated in the all 3-time experiment. The dot indicates significance at *P*<.05. (B) Averaged 3-month correlation between PSQI and relative power in difference frequencies in open-eye and closed-eye states. The dot indicates significance at *P*<.05. (C) Scatter plot showing the relationship between low α power in both open-eye and closed-eye states and PSQI for participants who participated in all 3-time experiments in months 1, 2, and 3. (D) Scatter plot showing the relationship between medium α power in both open-eye and closed-eye states and PSQI for participants who participated in all 3-time experiments in months 1, 2, and 3. (E) Scatter plot showing the relationship between high α power in both open-eye and closed-eye states and PSQI for participants who participated in all 3-time experiments in months 1, 2, and 3. PSQI: Pittsburgh Sleep Quality Index.

**Figure 5:**
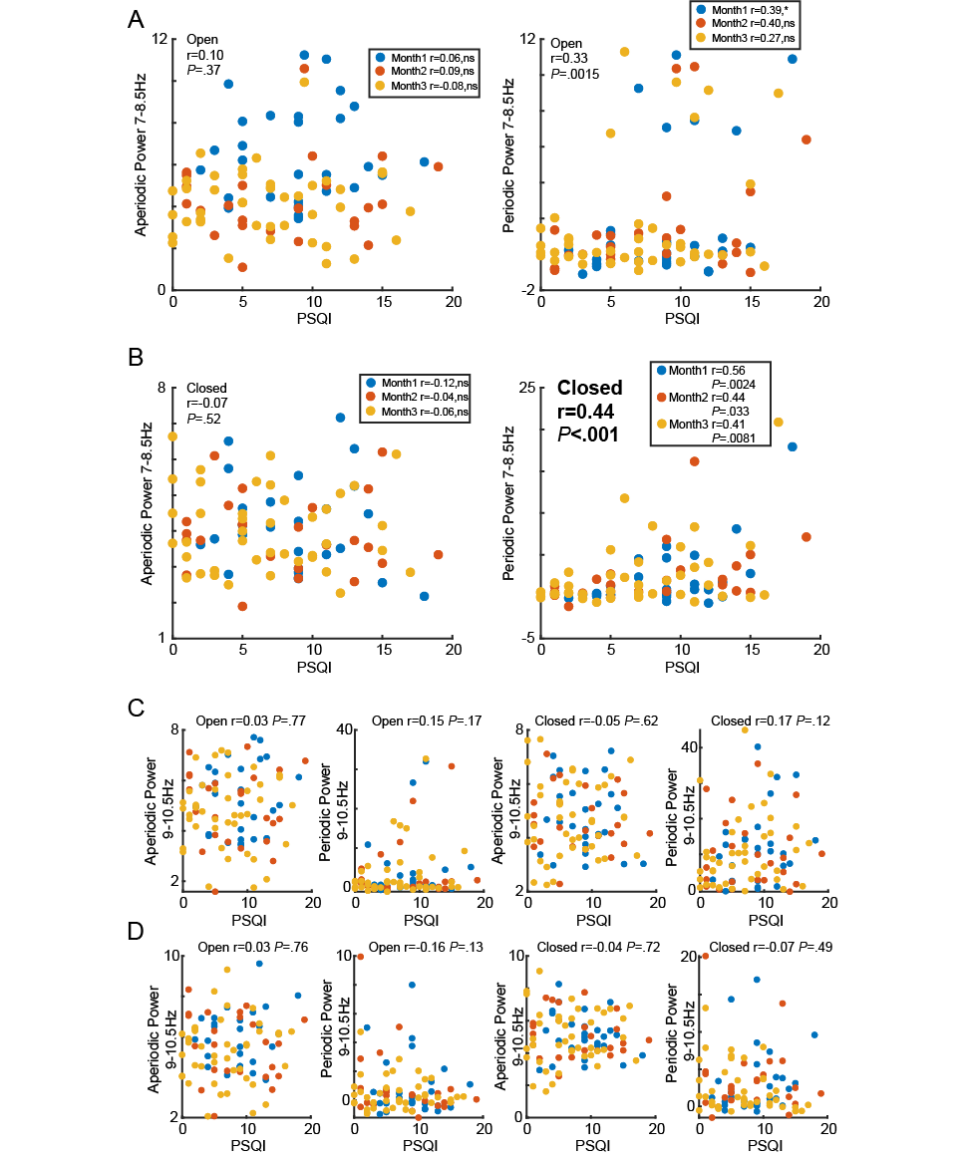
Relationship between PSQI and strength of sub-α power in aperiodic and periodic activity for all participants. (A) Scatter plot showing the relationship between low α power of aperiodic and periodic activity in the open-eye state and PSQI for all participants in months 1, 2, and 3. (B) Scatter plot showing the relationship between low α power of aperiodic and periodic activity in the closed-eye state and PSQI for all participants in months 1, 2, and 3. (C) Scatter plot showing the relationship between medium α power of aperiodic and periodic activity in open-eye and closed-eye states and PSQI for all participants in months 1, 2, and 3. (D) Scatter plot showing the relationship between high α power of aperiodic and periodic activity in open-eye and closed-eye states and PSQI for all participants in months 1, 2, and 3. PSQI: Pittsburgh Sleep Quality Index.

**Figure 6:**
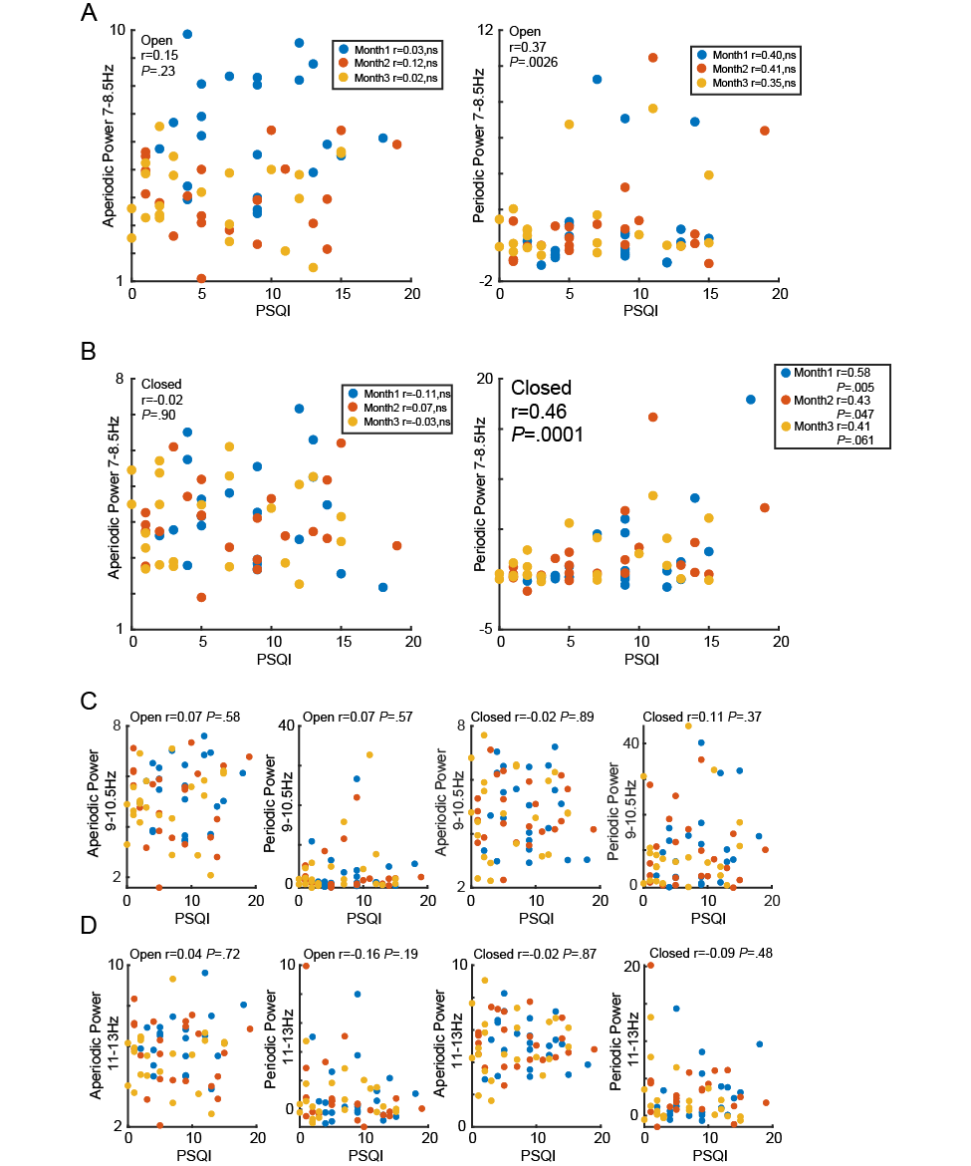
Relationship between PSQI and strength of sub-α power in aperiodic and periodic activity for participants who participated in the all 3-time experiment. (A) Scatter plot showing the relationship between low α power of aperiodic and periodic activity in the open-eye state and PSQI for participants who participated in all 3-time experiments in months 1, 2, and 3. (B) Scatter plot showing the relationship between low α power of aperiodic and periodic activity in closed-eye states and PSQI for participants who participated in all 3-time experiments in months 1, 2, and 3. (C) Scatter plot showing the relationship between medium α power of aperiodic and periodic activity in open-eye and closed-eye states and PSQI for participants who participated in all 3-time experiments in months 1, 2, and 3. (D) Scatter plot showing the relationship between high α power of aperiodic and periodic activity in open-eye and closed-eye states and PSQI for participants who participated in all 3-time experiments in months 1, 2, and 3. PSQI: Pittsburgh Sleep Quality Index.

In Figures 1B and 1E, a pairwise *t* test was examined to test the differences in relative α power between open-eye and closed-eye states in 3 months respectively. Similarly, in Figure 2C, a pairwise *t* test was used to test the differences in relative α power associated with aperiodic activity between these states over the same time period. In Figure 2D, a pairwise *t* test was used to evaluate the differences in relative α power related to periodic activity between open-eye and closed-eye states in 3 months respectively. In Figures 1C and 1F, a *t* test with Bonferroni correction was used to compare the differences in relative α power among 3 months for both open-eye and closed-eye states. Additionally, in Figures 3A and 3B and Figures 4A and 4B, Pearson correlation analysis was conducted to measure the relationship between relative power at each frequency and the PSQI. Figures 3C-E and 4C-E display Pearson correlations between PSQI and relative power in low α (LA, 7-8.5 Hz), medium α (MA, 9-10.5 Hz), and high α (HA, 11-13 Hz) bands, respectively. Figures 5 and 6 illustrate the Pearson correlation between PSQI and relative power in aperiodic and periodic activity across the LA, MA, and HA bands, respectively.

## Results

### Overview

In this study, we aimed to explore the relationship between different frequency components of portable EEG and sleep quality, and whether a stable neurobiological feature strongly associated with sleep quality can be found through portable EEG. First, we collected a batch of portable EEG data from participants in the first month and identified some features as potential targets that are significantly correlated with sleep quality. However, it cannot be determined whether this result can be repeated and stable. Therefore, we collected 2 additional batches of data in the second and third months, with 22 participants from the 2 follow-up periods also participating in the first data collection. Through this, we verified that the significant relationship between the target feature and sleep quality can be repeated and stable. During these 3 months of data collection, other participants also participated in the data collection.

### Stable Neural Recording Using Portable EEG Device in 3 Months

Our data collection lasted for 3 months, with EEG data collected once each month and the PSQI scale used to assess sleep quality. Before conducting the correlation analysis, we needed to verify the signal quality of the portable EEG device, which had 2 recording electrodes in the prefrontal region (FP1 and FP2). We performed frequency analysis on the EEG data collected each month (in both open-eye and closed-eye conditions), and after averaging at the group level, we observed an obvious peak around 10 Hz (FP1, Figure 1A; FP2, Figure 1D), representing α oscillations. The spectral shapes were similar, and the α oscillation intensity in the closed-eye condition was significantly stronger than in the open-eye condition across all 3 months (*P*<.001; FP1, Figure 1B; FP2, Figure 1E), which is consistent with previous research [54-57]. The relative power in the α band (8-13 Hz) showed no significant differences (*P*>.05) between open-eye and closed-eye conditions over the 3 months (FP1, Figure 1C; FP2, Figure 1F). Therefore, in subsequent analyses, we will average the spectra of the 2 prefrontal electrodes to represent the neural oscillatory activity in the prefrontal region.

### Low α-Band Activity Significant Correlated With Sleep Quality

After ensuring the stability of the portable EEG signal recordings, we first performed a correlation analysis between relative power across different frequency bands (5-30 Hz) and PSQI (Figure 3A, with the first month represented in blue, the second in orange, and the third in yellow). Although significant correlations appeared in multiple frequency bands each month (as indicated by the solid-colored dots), one band consistently showed a significant positive correlation across all 3 months, specifically the LA band (around 7-8.5 Hz), both in open-eye (Figure 3A left) and closed-eye (Figure 3A right) conditions. When we averaged the correlation coefficients across the 3 months, this phenomenon became even more pronounced (Figure 3B).

Based on previous studies [54,58,59], the traditional α band (8-13 Hz) may contain several suboscillations, such as LA, MA, and HA. In the subsequent analysis, we fixed 3 frequency bands to correspond to LA (Figure 3C), MA (Figure 3D), and HA (Figure 3E) to explore the relationship between the relative energy of these α subbands and sleep quality. We found a significant positive correlation between the relative power in the LA band and PSQI, a result that did not appear in the MA and HA bands. This significance was stronger in the closed-eye condition (*r*=0.45, *P*<.001) than in the open-eye condition (*r*=0.26, *P*=.01).

Since the number of participants varied each month, Figure 4 presents a generalized group-level result across a broad population. However, 22 participants in our data set completed all 3 experiments, allowing us to examine whether this LA biomarker remains consistent within the same group. The results from this consistent group (Figure 4) were also aligned with those of the broader population. Our findings suggest that the increase in the relative power within the LA band is significantly correlated with a decline in sleep quality, a result that remained consistent across multiple follow-ups in a broad population.

### Periodic Activity in LA Band Contributes to the Relationship Between

Furthermore, to more precisely characterize the different components in the spectrum, we used a classic descriptive mathematical model to decompose each participant’s spectral data for each recording into periodic activity (Figure 2A) and aperiodic activity (Figure 2B). After decomposition, we found no significant differences in aperiodic activity between open-eye and closed-eye conditions (Figure 2C); the differences were primarily observed in the periodic activity (Figure 2D). These comparisons remained consistent across the 3 months of recordings (Figure 2A-D).

The next question we addressed was whether the significant correlation between sleep quality and LA intensity shown in the previous results was mainly contributed by the periodic or aperiodic components (Figure 5). After the decomposition, we plotted scatterplots to show the correlations between PSQI and both periodic and aperiodic activities in the LA, MA, and HA bands. We found that, in the LA band, periodic activity was significantly correlated with PSQI (*P*<.01) in both open-eye and closed-eye conditions, while aperiodic activity was not. In the MA and HA bands, neither periodic nor aperiodic activities were significantly correlated with PSQI (*P*>.05).

Additionally, the results from the consistent group of participants (Figure 6) were aligned with those from the broader population (Figure 5). Our findings suggest that the increase in periodic activity in the LA band is significantly associated with a decline in sleep quality, a result that remained consistent across multiple follow-ups in a broad population.

## Discussion

### Principal Results

Rapid screening for cognitive functions, such as sleep quality, requires identifying stable and reliable neurobiological targets. Additionally, for convenience, portable EEG devices are essential for quick data collection. In this study, we explored the relationship between the neurobiological characteristics of portable EEG and sleep quality through multiple follow-ups for the first time. We found that, at the group level, the relative power in the LA band of the prefrontal region was significantly positively correlated with recent sleep quality. More importantly, this correlation remained consistent after several months of continuous follow-up, regardless of whether we considered the same population or an expanded cohort. Furthermore, through detailed decomposition of the spectrum, we discovered that the periodic components of the LA band primarily contributed to this significant correlation. Our research lays a solid foundation for using portable EEG for rapid screening of sleep-related issues, bringing this technology into households everywhere in the future.

### Comparison With Prior Work

Sleep is a classic issue in the field of neuroscience [60-64]. During sleep, the EEG signals show specific patterns in different stages. In the beginning, high-frequency (β, γ, and α), low-voltage waves will gradually switch to higher voltage, slower waves (θ and δ), which are the feature patterns of nonrapid eye movement (NREM) sleep [18,20,65]. During NREM sleep, stages 1, 2, and 3 are classified depending on the EEG frequency and specific pattern. Spindles or bursting firing of neurons were found shortly after falling asleep. During rapid eye movement sleep, the EEG patterns were similar to those during awake [18]. In rodent studies, compared with humans, rats exhibit a more fragmented sleep pattern, with most research primarily focusing on rapid eye movement and NREM sleep stages [9,24]. During deep sleep, δ band activity often predominates, while during light sleep, θ oscillations are more prevalent. α activity typically appears in the resting state with eyes closed before sleep or during drowsiness with eyes open [66,67], which suggests that α oscillations themselves are a prelude to the sleep phase. If there is an abnormal α oscillation in the brain, it is likely to affect sleep [68,69], particularly during the process of falling asleep. However, it remains unclear how different sub-α oscillations are related to sleep quality, and this is the basis of our research. Therefore, our study does not focus on changes in neural oscillations during sleep but instead examines whether resting-state EEG has oscillatory characteristics that are significantly and consistently associated with subjective sleep quality. One possible intervention for improving sleep is yoga or meditation training, which has been shown in previous studies to significantly enhance sleep quality [70,71] and is also closely related to α oscillations [72]. Our research further refines this relationship by finding that, among various sub-α oscillations, LA band activity is significantly negatively correlated with sleep quality at the group level, while no such correlation was found with medium or HA band activity. This result could also be applied to the field of neuromodulation in the future. Improving sleep quality is a concern for many people, and previous studies have found that techniques like TACS can enhance sleep quality [65,73-75]. However, a fundamental question is how to select the appropriate frequency band for stimulation. Our research will provide important insights in this regard.

### Mechanisms of Suboscillations in α Band Activity

Our study focused primarily on the α frequency band, as it is widely recognized in EEG research as a prominent biomarker with strong underlying neurobiological mechanisms [76-79]. However, our analysis also included other frequency bands, such as θ and β (Figures 3A and 4A). These bands, however, did not show stable and consistent significant correlations with sleep quality. Although the 8-13 Hz α oscillation band is narrow, it includes various suboscillations. Some studies suggest that these α suboscillations may have different origins [58,59] and are associated with different cognitive functions [80,81], but the finer neural mechanisms underlying these suboscillations remain unclear. From a dynamical systems perspective [82], the generation of different α suboscillations could be related to the differing time constants of excitatory and inhibitory neurons within the system, directly leading to frequency changes. This implies that different α suboscillations may have hierarchical relationships. It may also explain the discrepancies in previous studies regarding the mechanisms of α oscillations. For instance, some believe they originate subcortically (such as the pulvinar thalamus [83-86]), while others argue for a cortical origin (such as layer 5 of the cortex [87,88]). Our study used electrodes to record from the prefrontal cortex, but due to the relatively low spatial resolution of EEG, we cannot guarantee that the signal originates solely from the prefrontal cortex. It could also come from other brain regions or nuclei. Future studies will need to design more experiments to verify the relationship between different α suboscillations and various brain regions.

### Potential Applications on Large-Scale Screening of Sleep Disorders in the Population

Currently, compared with other methods for capturing brain neural responses (functional magnetic resonance imaging, functional near-infrared spectroscopy, magnetoencephalography, and various invasive recording methods), functional magnetic resonance imaging and magnetoencephalography are expensive and have strict use conditions, while functional near-infrared spectroscopy lacks good spatial and temporal resolution. The most likely method to be widely applied in human communities is portable EEG. Although it does not have good spatial resolution, its temporal resolution and ability to reliably capture electrical signals make it particularly effective. This is especially true for the precise detection of α oscillations, which can be correlated with cellular-level or local circuit level of α oscillation mechanisms observed in animal experiments [89,90]. Just as we hoped for a rapid screening method to detect COVID-19 infections during the pandemic [48,91], today, we also desire a rapid screening tool for detecting cognitive function abnormalities in large populations. The results from current portable EEG research happen to meet this need, and our study provides strong support for this approach. In the future, we will design more experiments related to cognitive functions to fully leverage the advantages of portable EEG.

### Limitations

One limitation of this study is that it only explored the relationship between the prefrontal region and sleep quality. However, other brain regions may also be associated with sleep quality. Due to the limited number of electrodes in the portable EEG system, we were unable to address this question in this study. Therefore, in future experiments, we plan to develop portable EEG systems with more electrodes to further investigate this issue. Another issue is that there are various types of artifacts in EEG data. Since we are using a portable EEG device, efforts have been made to make it as portable as possible, which resulted in a reduction in the number of electrodes used. The device only has 2 electrodes, and therefore, the EEG data from this device is not suitable for traditional artifact removal methods, such as principal component analysis or independent component analysis [92,93]. We acknowledge that there may be potential muscle artifacts in the FP1 and FP2 channels, but these artifacts primarily affect the β or γ frequency bands. Our study, however, specifically avoids these controversial frequency bands and focuses solely on the α band, which is safe and uncontroversial. One more issue is that the source of EEG signals has always been a topic of debate [94,95]. While we observed this phenomenon in the prefrontal cortex, we cannot currently determine whether it originates solely from the prefrontal region without the involvement of other brain areas, this question could potentially be addressed by combining intracranial EEG with surface EEG for source localization. Last, this study does not argue that abbreviated analyses should replace more detailed methods universally, but rather that they offer a viable alternative for certain types of studies, especially those that prioritize efficiency. A deeper, more comprehensive analysis of EEG data can yield valuable insights as well, the trade-offs study between abbreviated and more comprehensive EEG analysis methods should be further compared in the future.

### Conclusions

These findings mark the first identification of a stable and reliable neurobiological target—relative power in the LA band during the closed-eye state—associated with sleep quality across multiple follow-up sessions. Our results lay a robust foundation for future applications of portable EEG devices in monitoring sleep quality and screening for sleep disorders across diverse populations.

## Data Availability

The datasets generated or analyzed during this study are not publicly available due to the policy of the institution but are available from the corresponding author on reasonable request.

## Abbreviations

EEG: electroencephalogram
HA: high α
LA: low α
MA: medium α
NREM: nonrapid eye movement
PSQI: Pittsburgh Sleep Quality Index
